# Preparation of Hydrated TiO_2_ Particles by Hydrothermal Hydrolysis of Mg/Al-Bearing TiOSO_4_ Solution

**DOI:** 10.3390/nano13071179

**Published:** 2023-03-25

**Authors:** Shuyu Lin, Fan Yang, Zhuoying Yang, Jing Wang, Lan Xiang

**Affiliations:** 1 Department of Chemical Engineering, Tsinghua University, Beijing 100084, China; 2 School of Chemical Engineering, Sichuan University, Chengdu 610065, China; 3 Key Laboratory of Synthetic and Biological Colloids, Ministry of Education, School of Chemical and Material Engineering, Jiangnan University, Wuxi 214122, China

**Keywords:** hydrated TiO_2_, TiOSO_4_, hydrothermal hydrolysis, MgSO_4_ and Al_2_(SO_4_)_3_

## Abstract

As the byproduct in the smelting process of vanadium titano-magnetite, titanium-bearing blast furnace slag (TBFS) can be converted to a titanyl sulfate (TiOSO_4_) solution containing MgSO_4_ and Al_2_(SO_4_)_3_ impurities via dissociation by concentrated H_2_SO_4_ (80–95%) at 80–200 °C, followed by leaching with H_2_O at 60–85 °C. In this study, hydrated TiO_2_ was prepared by hydrothermal hydrolysis of a Mg/Al-bearing TiOSO_4_ solution at 120 °C and the hydrolysis law was investigated. The experimental results indicate that MgSO_4_ and Al_2_(SO_4_)_3_ accelerated the hydrolysis and significantly affected the particle size (increasing the primary agglomerate size from 40 to 140 nm) and dispersion (reducing the aggregate size from 12.4 to 1.5 μm) of hydrated TiO_2_. A thermodynamic equilibrium calculation showed TiOSO_4_ existed as TiO^2+^ and SO_4_^2−^ in the solution, and MgSO_4_ and Al_2_(SO_4_)_3_ led to little change of [TiO^2+^], but an obvious decrease of [H^+^], which favored the hydrolysis process. At the same time, the coordination–dissociation mechanism of SO_4_^2−^ and Al(SO_4_)_2_^−^ facilitated the lap bonding of Ti-O-Ti, promoting the growth of hydrated TiO_2_ synergistically.

## 1. Introduction

China has abundant reserves of titanium resources ranking among the top in the world, nearly 92.4% of which are in the form of vanadium titano-magnetite in Panzhihua, Sichuan province [1]. These raw ores are usually employed for blast-furnace puddling, leaving most titanium in the residual slags, known as titanium bearing blast furnace slag (TBFS) [2]. TBFS contains 22–25% TiO_2_ and can be regarded, essentially, as a multi-component symbiotic low-grade titanium ore, which is a valuable and significant treasure for recycling. However, the comprehensive utilization ratio of it is less than 3% [3], and the only way to recycle it currently is to treat it as an admixture for cheap building materials, owing to its complicated natural structure and corresponding extraction technology obstacles [4]. Since the 1970s, the total accumulated amount of TBFS has reached approximately 80 million tons, with an annual dischargement over 3.8 million tons [5]. The stacking of TBFS encroaches on a large area of land and results in serious environmental pollution and a huge waste of titanium resources [6]. The utilization and upgradation of TBFS have become a worldwide problem and have aroused extensive concern.

As the third inorganic chemical product, TiO_2_ is promising in broad applications, such as pigment, dye-sensitized solar cell (DSSC), light degradation and so on, for its outstanding properties [7]. Particularly, it has excellent UV absorption performance, which can effectively shield UV rays and prevent paint or plastic from aging and becoming discolored after long-term exposure to the sun, and has an irreplaceable position in architectural paint, wallpaper, coatings, etc. Extracting and preparing pigment-grade TiO_2_ from TBFS can not only solve the problem of waste and pollution, but also bring huge economic benefits, which is of great significance to the efficient utilization of resources and sustainable development. However, the Ti-containing minerals in TBFS interconnect with each other, leading to a complex embedding structure, and the application scenarios of pigments strictly require the appropriate granularity (around 230 nm) and high dispersion of TiO_2_, which brings great challenges to the preparation technology. For this vision, many processing techniques have been carried out, such as the high-temperature carbonization and low-temperature chlorination method, the H_2_SO_4_ method, the HCl method and the NaOH molten salt roasting method [8,9,10]. Among them, the H_2_SO_4_ method has been most widely used to prepare TiO_2_ in industry and provides an available path for the reuse of TBFS due to its low cost, simple and mature preparing process, and so forth [11]. In this method, a TiOSO_4_ solution is first prepared by acidolysis of TBFS with concentrated H_2_SO_4_ at 80–200 °C, and then inoculated with anatase seeds under a slight boiling state to initiate the hydrolysis and prepare hydrated TiO_2_ (TiO_2_·H_2_O, metatitanic acid), which is an important intermediate product and can convert to TiO_2_ over 700 °C [12,13].

As for the whole process, the hydrolysis of TiOSO_4_ is the key step in TiO_2_ production and will ultimately determine the morphology, dispersion and performance of the final product [14]. The hydrolysis reaction of TiOSO_4_ has been widely studied, and many researchers have worked on the influencing factors of this reaction, such as the titanium and acid concentration, reaction temperature, crystal seed and stirring speed [15,16,17,18]. However, TBFS is a multi-element complex byproduct in the ironmaking process, and the Mg and Al elements therein will enter the solution together with Ti in the form of MgSO_4_ and Al_2_(SO_4_)_3_ during the acidolysis process [19], the effect of which on the hydrolysis has been seldom studied. At the same time, less Ti content in TBFS results in a very low TiOSO_4_ concentration (0.8 mol·L^−1^) in the hydrolysis solution, which is only about a quarter of the industrial TiOSO_4_ concentration and will lead to serious agglomerate, coarse and uneven hydrated TiO_2_, harming the final properties.

The formation of hydrated TiO_2_ is mainly through the polycondensation reaction of Ti complexes, namely, hydroxy-bridging and oxy-bridging. The cleavage of Ti-OH_2_ follows the SN1 mechanism. The titanium complexes form a hydroxy-bridge transition state by nucleophilic addition reaction and 1,3-proton transfer, and then deprotonate to form a thermodynamically stable oxygen bridge. Since proton transfer often requires high activation energy, the hydroxy-bridging process is slow. The hydrothermal method is a simple, efficient, harmless synthesis method, which is widely used in the preparation of various metal oxides, such as TiO_2_ nanoparticles, ZnO whiskers, Cu_2_O hollow spheres, etc. [20,21,22]. Under a hydrothermal condition, the solution has relatively low viscosity and large density change, which makes the solution have greater convection driving force and stronger ion diffusion, accelerating the reaction. The increase of the hydrothermal temperature can effectively promote the hydrolysis and polycondensation of metal cations, thus regulating the growth and aggregation behavior of hydrated TiO_2_. The powder obtained by this method has the advantages of complete grain, small particle size, uniform distribution and light particle agglomeration. For these reasons, the hydrothermal method is expected to be used in the TiOSO_4_ solution hydrolysis system to prepare hydrated TiO_2_ with intact morphology.

Herein, we successfully achieved the rapid and complete hydrolysis of a Mg/Al-bearing low concentration TiOSO_4_ solution by a low-temperature hydrothermal method, and prepared hydrated TiO_2_ with a uniform particle size and homogeneous morphology. The effect of MgSO_4_ and Al_2_(SO_4_)_3_ on the hydrolysis reaction was studied. The experiments and thermodynamic calculations confirmed that MgSO_4_ and Al_2_(SO_4_)_3_ could accelerate the hydrolysis of TiOSO_4_ and promote the growth of hydrated TiO_2_ primary agglomerates, which was speculated to be related to the association of sulfate with H^+^ and Ti complexes.

## 2. Materials and Methods

### 2.1. Synthesis of Hydrated TiO_2_

The TBFS was obtained from Pangang Group Company Limited (Sichuan, China) with a chemical composition (calculated as oxides) as shown in Table 1. For the H_2_SO_4_ method, the TBFS was first converted to a sulfate mixture in concentrated H_2_SO_4_ (80–95%) at 80–200 °C. The soluble components were then fully leached from the reacted slag with H_2_O at a temperature of 60–85 °C to obtain the Mg/Al-bearing TiOSO_4_ solution. According to the chemical composition of the leaching solution, appropriate amounts of TiOSO_4_ (Shanghai Macklin Biochemical Co., Ltd., Shanghai, China), Al_2_(SO_4_)_3_·18H_2_O (Shanghai Macklin Biochemical Co., Ltd., Shanghai, China), MgSO_4_ (Sinopharm Chemical Reagent Co., Ltd., Beijing, China) and 98% H_2_SO_4_ (Beijing Tongguang Fine Chemical Co., Ltd., Beijing, China) were dissolved in water at 50 °C and stirred until completely clear to obtain the simulated Mg/Al-bearing TiOSO_4_ solution, the chemical composition of which is presented in Table 1.

A total of 50 mL of the simulated Mg/Al-bearing TiOSO_4_ solution was transferred to a 100 mL Teflon-lined stainless-steel autoclave (Beijing xingde instrument equipment Co., Ltd., Beijing, China). The autoclave was sealed and fixed in a homogeneous reactor (Songling Chemical Equipment Co., Ltd., Yantai, China) and rotated around the axis at a speed of 60 rpm at 120 °C. The reaction product was centrifuged at 3000 rpm for 3 min, and the supernatant was used to detect the hydrolysis ratio. White precipitates were obtained via filtration of the suspension and afterwards washed with 100 mL 5% diluted H_2_SO_4_ solution and deionized water three times. The solid was then dried at 100 °C for 4 h to obtain hydrated TiO_2_. All the chemicals were of analytical-grade purity and the deionized water with a resistivity >18 MΩ·cm^−1^ was used throughout the whole experiment.

### 2.2. Characterization

The chemical compositions of the supernatant and the Mg/Al-bearing TiOSO_4_ solution were analyzed by inductively coupled plasma optical emission spectroscopy (ICP-OES, Spectro Arcos, Spectro, Kleve, Germany) with a standard titanium solution (1000 μg·mL^−1^, HNO_3_) as a reference. Phase identification and crystallite size measurement of the hydrated TiO_2_ were carried out using an X-ray diffractometer (D8 Advance, Bruker, Karlsruhe, Germany) with Cu Kα (λ = 0.154178 nm) radiation. The crystallite average size (d) was determined based on Scherrer’s equation: $d=\frac{\lambda K}{\beta \cos\theta }$, where *λ* is the wavelength of Cu Kα radiation, K is the particle shape factor and *β* is the full width at half maximum of the intensity peak. The average size of the crystallites was calculated from the (101) reflection of anatase TiO_2_, and the calculation of the half-peak width took the so-called device broadening into account. A field emission scanning electron microscope (FESEM, JSM 7401F, JEOL, Tokyo, Japan) and a high-resolution transmission electron microscope (HRTEM, JEM-2010, JEOL, Tokyo, Japan) were used to investigate the morphology and microstructure and measure the primary agglomerate particle size of hydrated TiO_2_. The average aggregate size was determined by a Malvern laser particle size analyzer (LPSA, Mastersizer3000, Malvern, UK). The elemental species and chemical states on the sample surface were characterized by an X-ray photoelectron spectrometer (XPS, PHI-5300, PHI, Lafayette, LA, USA). Fourier transform infrared spectra of the hydrated TiO_2_ were recorded using a Fourier transform infrared spectrophotometer (FT-IR, IRTracer-100, SHIMADZU, Kyoto, Japan). In addition, the samples were subjected to the thermogravimetric analysis (TGA, DSC1/1100, METTLER TOLEDO, Zurich, Switzerland) under a N_2_ atmosphere with temperatures ramped from 30 °C to 1000 °C at 10 °C·min^−1^.

## 3. Results and Discussion

### 3.1. Characterization of Hydrated TiO_2_

The hydrothermal hydrolysis reaction of TiOSO_4_ is shown in Equation (1). The nucleation and growth of hydrated TiO_2_ involves three length-scale particles, i.e., crystals, primary agglomerates and aggregates. With the hydrolysis of TiOSO_4_, the small crystal grains overlap to form primary agglomerates, which are uniform and narrow in size. Numerous primary agglomerates further reunite to obtain aggregates, constituting hydrated TiO_2_.

(1) $$TiO^{2+}+2H_{2}O=TiO_{2}\cdot H_{2}O↓+2H^{+}$$

**Figure 1:**
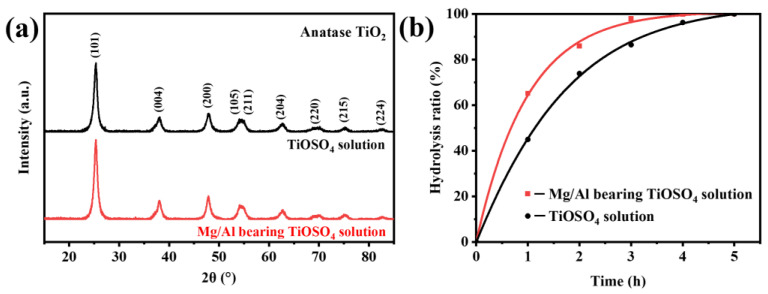
(**a**) XRD patterns of hydrated TiO_2_; (**b**) Hydrolysis ratio curves.

The phase and grain size of hydrated TiO_2_ hydrothermally precipitated from the TiOSO_4_ solutions with and without MgSO_4_ and Al_2_(SO_4_)_3_ were first determined by X-ray diffraction analysis, respectively. As Figure 1a shows, the XRD peaks of both samples are well indexed to anatase TiO_2_ (PDF 99-0008). The characteristic peak intensities of the two samples are almost the same, but the half-peak widths of hydrated TiO_2_ obtained from the Mg/Al-bearing TiOSO_4_ solution are narrower. The average crystallite size was calculated to be 9.3 nm based on the strongest peak of the (010) crystal surface diffraction, 0.3 nm lager than that of the TiOSO_4_ solution.

**Figure 2:**
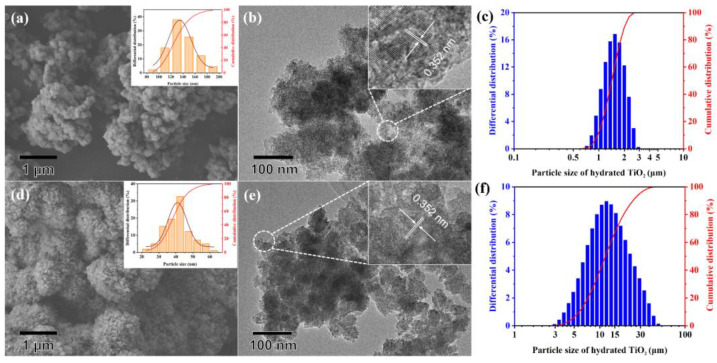
(**a**) SEM of hydrated TiO_2_ prepared from the Mg/Al-bearing TiOSO_4_ solution with an illustration of the primary agglomerate particle size distribution; (**b**) TEM of hydrated TiO_2_ prepared from the Mg/Al-bearing TiOSO_4_ solution; (**c**) Aggregate particle size distribution for the Mg/Al-bearing TiOSO_4_ solution; (**d**) SEM of hydrated TiO_2_ prepared from the TiOSO_4_ solution with an illustration of the primary agglomerate particle size distribution; (**e**) TEM of hydrated TiO_2_ prepared from the TiOSO_4_ solution; (**f**) Aggregate particle size distribution for the TiOSO_4_ solution.

As shown in Figure 2, MgSO_4_ and Al_2_(SO_4_)_3_ have a significant influence on both the primary agglomerate and dispersion of hydrated TiO_2_. The primary agglomerates deposited from the TiOSO_4_ solution (Figure 2d) show a regular spherical morphology with clear edges, but a serious and uneven agglomeration behavior. The smallest agglomerate is 3 μm, while the largest one reached 20 μm with an extremely irregular shape. In contrast, the primary agglomerates for the Mg/Al-bearing TiOSO_4_ solution (Figure 2a) are significantly larger with blurred boundaries, and their aggregates are spherical or ellipsoid with good dispersion. The particle size statistical results presented the primary agglomerate particle distribution for the Mg/Al-bearing TiOSO_4_ solution (illustration in Figure 2a) is 80–200 nm and the average size is close to 140 nm, which reaches three times that of the TiOSO_4_ solution (average size around 40 nm). The lager specific surface area considerately reduces the surface energy, which increases dispersion (Figure 2c,f). Their average aggregate sizes are 1.5 and 12.4 μm, corresponding to the Mg/Al-bearing TiOSO_4_ solution and the TiOSO_4_ solution, respectively. Additionally, the two samples have a similar grain size, but the grain orientation for the TiOSO_4_ solution is more disordered (Figure 2b,e). The lattice spacing of 0.352 nm observed in the HRTEM images of both samples is attributed to the (101) plane of the TiO_2_, which is consistent with the XRD results.

**Figure 3:**
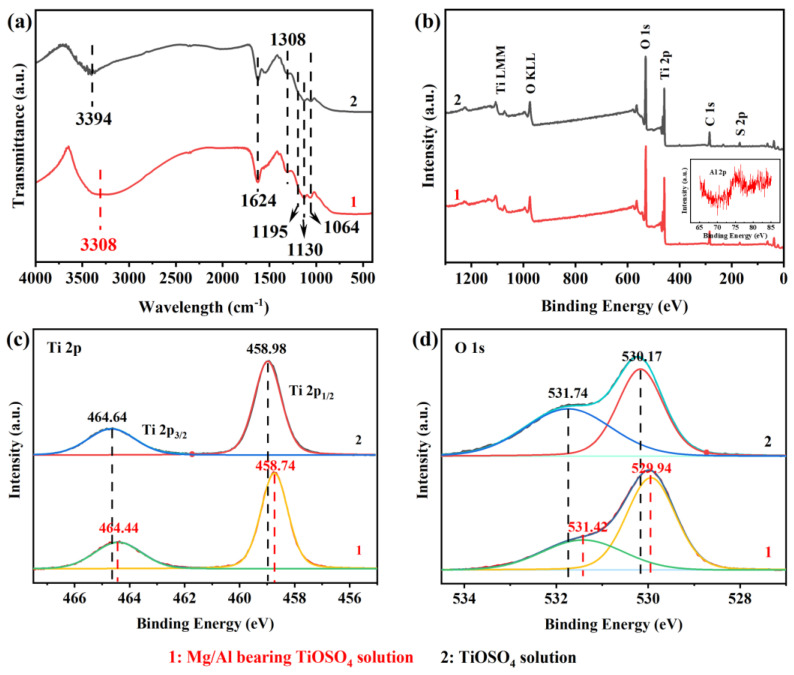
The (**a**) FT-IR patterns, (**b**) overall XPS (with an insert figure of Al 2p XPS spectra), (**c**) Ti 2p XPS and (**d**) O 1s XPS spectra of hydrated TiO_2_ in the Mg/Al-bearing TiOSO_4_ solution and the TiOSO_4_ solution.

To explore the reasons for the significant effect of MgSO_4_ and Al_2_(SO_4_)_3_ on the hydrated TiO_2_, the surface features of the samples were characterized by FT-IR and XPS. As shown in Figure 3a, a broad absorption peak at about 3300 cm^−1^ attributed to the stretching of the -OH bond and a strong absorption peak at 1624 cm^−1^ due to H-O-H bending vibration appear in both samples, indicating the existence of surface hydroxyl groups and adsorbed water. A red shift from 3394 to 3308 cm^−1^ and a significantly increased integrated intensity of the -OH bond indicate the increased hydroxyl group and the decrease of the -OH bond force constant with the addition of MgSO_4_ and Al_2_(SO_4_)_3_. The characteristic peaks at 1308 cm^−1^, 1195 cm^−1^, 1130 cm^−1^ and 1064 cm^−1^ caused by O=S=O antisymmetric stretching vibration and O-S-O symmetric stretching vibration confirm the presence of SO_4_^2−^ on the surface. Meanwhile, no strong absorption band is found near 1370 cm^−1^, indicating that SO_4_^2−^ exists in the form of bidentate ligand rather than metal sulfate [23,24,25]. The overall XPS spectrum (Figure 3b) shows no peak of Mg, indicating that Mg did not exist on the surface of the hydrated TiO_2_. However, a weak peak of Al 2p was observed at 74.84 eV, even after the sample was fully washed, which proves the strong bonding between Al and the hydrated TiO_2_. The deconvoluted results (Figure 3c) present two peaks at 458.74 and 464.44 eV, attributed to Ti 2p_1/2_ and 2p_3/2_. Their shift towards low binding energy indicates a higher electron density of hydrated TiO_2_ for the Mg/Al-bearing TiOSO_4_ solution. The strong shoulder peak in Figure 3d at 529.94 eV is attributed to the Ti-O-Ti lattice oxygen, while the weak one at 531.42 eV is due to the surface hydroxyls and adsorbed water. The higher intensity of the strong shoulder peak and lower intensity of the weak one proves that the hydrated TiO_2_ has more lattice oxygen, less surface hydroxyl and adsorbed water in the Mg/Al-bearing TiOSO_4_ solution and the latter is related to the smaller surface area (Figure 2a). The offset of two peaks confirms a higher electron density for both the lattice and surface oxygen.

Combined with the results above, we can infer that MgSO_4_ and Al_2_(SO_4_)_3_ promote the removal of H^+^ from the titanium coordinated water, which is beneficial to the dissociation of water in the first stage and formation of the Ti-O-Ti bond by hydroxyl bridge dehydrogenation, thereby increasing the electron density of the coordination centers (Figure 3c,d). The prolonged Ti-O-Ti-O-Ti zigzag chain increases the steric hindrance of the hydroxyl group, which in turn reduces the -OH bond force constant and shifts the absorption peak to a lower wavelength (Figure 3a). Meanwhile, the bidentate chelate structure of sulfate between the titanium complexes may also play a positive role in this process.

### 3.2. Effect of MgSO_4_ and Al_2_(SO_4_)_3_ on the Hydrolysis Rate

To further demonstrate that MgSO_4_ and Al_2_(SO_4_)_3_ can promote H^+^ transfer and thus Ti-O-Ti bonding and hydrolysis of TiOSO_4_, the hydrolysis ratio curves were determined. Figure 1b shows the hydrolysis ratio curves of two solutions reacted at 120 °C for 1–5 h. The Boltzmann equation is one of the most typical sigmoidal curves, and the shape of its right half matches the TiOSO_4_ hydrolysis kinetic curve well. The Boltzmann function is given by:

(2) $$\alpha =\alpha_{0}+\frac{\alpha_{\max}-\alpha_{0}}{1+\exp[(t-t_{1/2})/\mathrm{dt}]}$$

where *α* is the hydrolysis ratio of TiOSO_4_ at time t; *α*_0_ and *α*_max_ represent the minimum and maximum value of the sigmoidal curve, respectively; t_1/2_ is the time when *α* = (*α*_0_ + *α*_max_)/2; and dt is a parameter describing the width of the curve along the time axis.

The fitting hydrolysis ratio curves are shown in Figure 1b. Both solutions were completely hydrolyzed under the hydrothermal condition of 120 °C within 5 h. However, the hydrolysis of the Mg/Al-bearing TiOSO_4_ solution is significantly faster than the pure TiOSO_4_ solution. The hydrolysis ratio of the former reached 65.2% after only 1 h and nearly 100% within 4.0 h, while for the latter, TiOSO_4_ was hydrolyzed only 44.9% after 1 h and completely after 5.0 h. The hydrolysis ratio gap between the two solutions first increased and then decreased, reaching a maximum of 20.2% at 1.0 h, which proves that MgSO_4_ and Al_2_(SO_4_)_3_ indeed promote the hydrolysis of TiOSO_4_, especially in the initial process.

### 3.3. Thermodynamic Calculation

#### 3.3.1. Reaction Equation for TiOSO_4_ Hydrothermal Hydrolysis System

**Figure 4:**
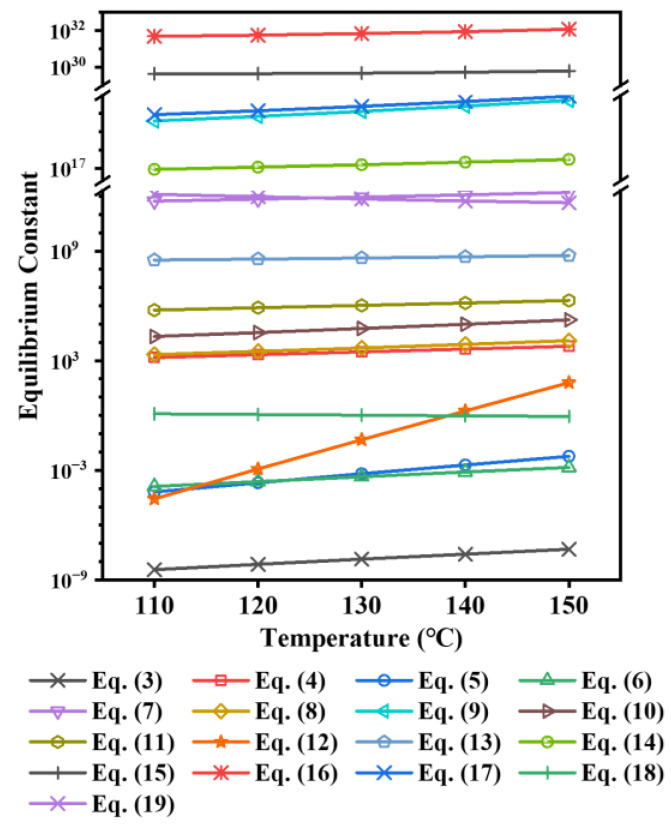
Reaction equilibrium constants.

In order to explore the main cause for the promoting effect of MgSO_4_ and Al_2_(SO_4_)_3_ on the hydrolysis of TiOSO_4_, the thermodynamic equilibrium calculation was carried out. The reaction equations involved in the Mg/Al-bearing TiOSO_4_ solution are listed in Equations (1) and (3)–(19). Those equations related to the TiOSO_4_ solution are Equations (1), (3), (4), (18) and (19). The thermodynamic data for all species are cited from reference [26], and the corresponding reaction equilibrium constants at 110–150 °C are shown in Figure 4.

(3) $$HSO_{4}^{-}+H^{+}=H_{2}SO_{4}$$

(4) $$SO_{4}^{2-}+H^{+}=HSO_{4}^{-}$$

(5) $$Mg^{2+}+SO_{4}^{2-}=MgSO_{4}(aq)$$

(6) $$Mg^{2+}+OH^{-}=Mg{\left(OH\right)}^{+}$$

(7) $$Mg^{2+}+2OH^{-}=Mg{\left(OH\right)}_{2}(aq)$$

(8) $$2Mg^{2+}+OH^{-}=Mg_{2}{\left(OH\right)}^{3+}$$

(9) $$4Mg^{2+}+4OH^{-}=Mg_{4}{\left(OH\right)}_{4}^{4+}$$

(10) $${\mathrm{Al}}^{3+}+SO_{4}^{2-}=AlSO_{4}^{+}$$

(11) $${\mathrm{Al}}^{3+}+2SO_{4}^{2-}=Al{\left(SO\right)}_{2}^{-}$$

(12) $${2\mathrm{Al}}^{3+}+3SO_{4}^{2-}=Al_{2}{\left(SO_{4}\right)}_{3}(aq)$$

(13) $${\mathrm{Al}}^{3+}+OH^{-}=Al{\left(OH\right)}^{2+}$$

(14) $${\mathrm{Al}}^{3+}+2OH^{-}=Al{\left(OH\right)}_{2}^{+}$$

(15) $${\mathrm{Al}}^{3+}+3OH^{-}=Al{\left(OH\right)}_{3}(aq)$$

(16) $${\mathrm{Al}}^{3+}+4OH^{-}=Al{\left(OH\right)}_{4}^{-}$$

(17) $${2\mathrm{Al}}^{3+}+2OH^{-}=Al_{2}{\left(OH\right)}_{2}^{4+}$$

(18) $$TiO^{2+}+SO_{4}^{2-}=TiOSO_{4}(aq)$$

(19) $$H^{+}+OH^{-}=H_{2}O$$

#### 3.3.2. Comparison of the Hydrolysis Tendency

**Figure 5:**
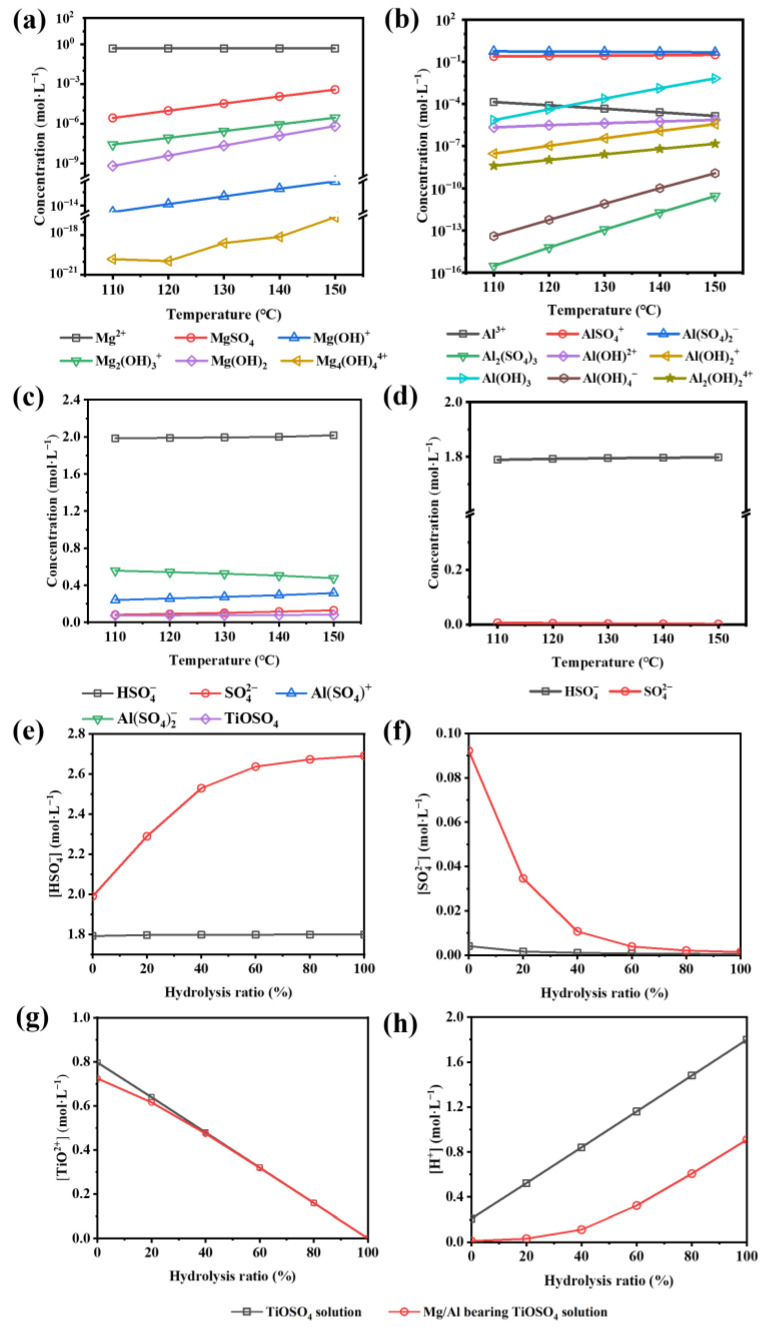
Initial equilibrium concentration: (**a**) Mg-containing components; (**b**) Al-containing components; (**c**) S-containing components in the Mg/Al-bearing TiOSO_4_ solution; (**d**) S-containing components in the TiOSO_4_ solution; the curves of the ion concentration with the hydrolysis ratio: (**e**) TiO^2+^; (**f**) H^+^; (**g**) HSO_4_^−^; (**h**) SO_4_^2−^.

The initial equilibrium states of the two solutions were firstly obtained by the Newton–Raphson iteration method to investigate the reasons for the considerable influence of MgSO_4_ and Al_2_(SO_4_)_3_ on the initial hydrolysis. As shown in Figure 5, the temperature has little effect on the initial equilibrium. At 110–150 °C, the magnesium-containing components mainly exist in the form of Mg^2+^ (Figure 5a). In contrast, Al does not exist independently in the solution, but forms complexes with SO_4_^2−^. The Al(SO_4_)_2_^−^ and AlSO_4_^+^ are the main ionic form of the Al-containing components, and the concentration of the former is about twice that of the latter (Figure 5b). The sulfur element mainly exists in the form of HSO_4_^−^ due to the strong acidity of both solutions (Figure 5c,d). For the TiOSO_4_ solution, there is almost no SO_4_^2−^ and the [HSO_4_^−^] is 1.79 mol·L^−1^ at 120 °C. In comparison, the [SO_4_^2−^] in the Mg/Al-bearing TiOSO_4_ solution is 0.09 mol·L^−1^, and the [HSO_4_^−^] is 1.99 mol·L^−1^, which is much higher than that of the TiOSO_4_ solution. The initial [TiO^2+^] and [H^+^] are most closely related to the hydrolysis and are shown in Figure 5e,f. The Mg/Al-bearing TiOSO_4_ solution has lower [TiO^2+^] and [H^+^] compared with the TiOSO_4_ solution.

With the hydrolysis of TiOSO_4_, TiO^2+^ forms Ti-O-Ti bonds with each other and continuously grows three-dimensionally, generating hydrated TiO_2_ and H^+^, breaking the original balance. According to Equation (2), an increase of [TiO^2+^] or a decrease of [H^+^] favors the hydrolysis. The initial [TiO^2+^] of the Mg/Al-bearing TiOSO_4_ solution was 0.72 mol·L^−1^, 0.08 mol·L^−1^ lower than that of the TiOSO_4_ solution (Figure 5e). However, the difference between the two gradually decreased with the hydrolysis and almost disappeared when the hydrolysis ratio reached 40%. Therefore, the promotion effect of TiO^2+^ is almost identical in the two solutions. At the same time, the [H^+^] in the Mg/Al-bearing TiOSO_4_ solution was always lower than that of the TiOSO_4_ solution (Figure 5f). The initial [H^+^] of the latter was 0.21 mol·L^−1^, while the former was only 0.01 mol·L^−1^, and the gap between them increased with the hydrolysis. The weaker inhibitory effect of H^+^ on the TiOSO_4_ hydrolysis in the Mg/Al-bearing TiOSO_4_ solution is the fundamental reason for its faster reaction rate. This is closely related with the change of S-containing components. As shown in Figure 5g,h, the [HSO_4_^−^] and [SO_4_^2−^] in the Mg/Al-bearing TiOSO_4_ solution were always higher than those in TiOSO_4_ solution. The S-containing components are mainly in the form of HSO_4_^−^, indicating that the strong association between SO_4_^2−^ and H^+^ greatly consumes H^+^, weakens the acidity of the solution and is beneficial to the hydrolysis. By the middle stage, the SO_4_^2−^ has been greatly converted to HSO_4_^−^ due to the generation of H^+^, and the [SO_4_^2−^] gap between the two solutions gradually decreases.

**Figure 6:**
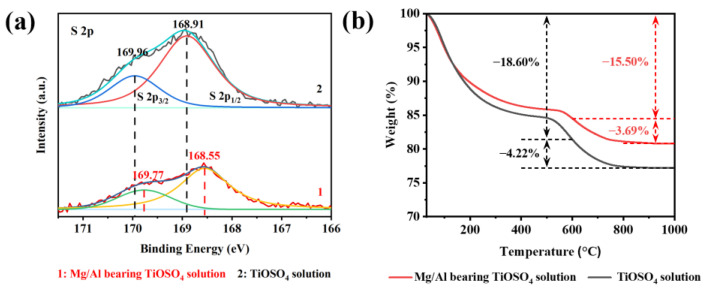
The (**a**) S 2p XPS and (**b**) TGA of hydrated TiO_2_ in the Mg/Al-bearing TiOSO_4_ solution and the TiOSO_4_ solution.

In addition to associating H^+^ with the reduction of the acidity of the solution, the effect of SO_4_^2−^ itself remains to be explored. Thermodynamic calculations show that, although most SO_4_^2−^ combines with H^+^ and converts to HSO_4_^−^, the remaining [SO_4_^2−^] still reaches a non-negligible order of magnitude compared with TiO^2+^. In Figure 6a, two characteristic peaks fitted by correlated Lorentzians at 168.91 (S 2p_1/2_) and 169.96 eV (S 2p_3/2_) prove the bidentate complexation between the SO_4_^2−^ and titanium complexes through the oxygen bridges, which fits with the FT-IR results. Such a structure will cause an electron distribution variation and positive charge excess in the side of S [27,28,29]. With the introduction of MgSO_4_ and Al_2_(SO_4_)_3_, the peaks move towards low binding energy, and the integral strength of both peaks decreases significantly, implying a reduction of the surface bidentate SO_4_^2−^, which is inseparable from the reduced specific surface area (Figure 2).

The bidentate SO_4_^2−^ ligands can be further divided into mononuclear and binuclear bidentate ligands and both may occur during hydrolysis. The former will occupy part of the titanium binding sites due to the steric hindrance, while the latter will significantly shorten the distance between the two titanium complexes, just like increasing their collision probability, thus facilitating the oxygen bridge bonding. As Figure 6b shows, the mass loss rates of the two samples at 30–600 °C are 18.60% and 15.50%, which are related to the release of free water (30–105 °C) and adsorbed water (105–600 °C). The mass loss of the hydrated TiO_2_ for Mg/Al-bearing TiOSO_4_ solution is 3.69% above 600 °C, which is attributed to the thermal decomposition of SO_4_^2−^ and is less than that of the TiOSO_4_ solution. An interesting phenomenon that was observed was that the hydrated TiO_2_ prepared from the solution with more SO_4_^2−^ contained less S. Accordingly, we can infer SO_4_^2−^ has the coordination–dissociation mechanism during the hydrolysis of TiOSO_4_, namely, SO_4_^2−^ can coordinate with one or two titanium complexes to form a reversible bidentate connection structure, which will not occupy the binding site of titanium complexes for a long time, but shorten the distance between them and promote the formation of the Ti-O-Ti bond and hydrolysis of TiOSO_4_.

Another interesting issue that cannot be ignored is the influence of cations on the hydrolysis of TiOSO_4_. According to the thermodynamic calculation results, Mg exists in the form of free ions in a solution, while the thermodynamic stable states of Al are Al(SO_4_)_2_^−^ and AlSO_4_^+^, which leads to another possibility. For Al(SO_4_)_2_^−^, the SO_4_^2−^ coordinated with Al still has negatively charged oxygen that can attack the titanium complex center to form an Al-O-S-O-Ti bond, which is consistent with the result of XPS (Figure 3b). The low strength of the peak indicates that this bond may be unstable or reversible, but its presence still plays a similar role to SO_4_^2−^, namely, pulling the titanium complex closer in the solution. However, due to the steric hindrance effect, its promotion on the formation of oxygen bridges is much weaker than that of the bidentate chelating structure of SO_4_^2−^. Tian et al. [30] have also reported the effect of Al: they found that Al^3+^ can affect the hydrolysis ratio at different temperatures and mentioned the adsorption between the Ti cluster and Al^3+^, but the simultaneous existence of SO_4_^2−^ and Cl^−^ made this question more complex. Additionally, we have not found an obvious intrinsic effect of Mg^2+^ on the hydrolysis of TiOSO_4_.

**Figure 7:**
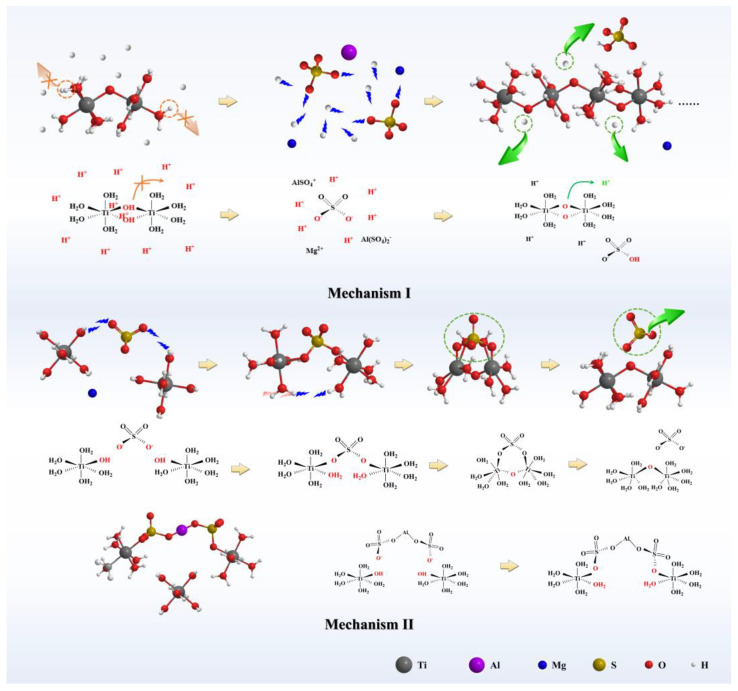
Influence mechanism of MgSO_4_ and Al_2_(SO_4_)_3_ on the hydrolysis of TiOSO_4_.

In summary, the influence of MgSO_4_ and Al_2_(SO_4_)_3_ on the hydrolysis of TiOSO_4_ can be attributed to the following points (Figure 7). MgSO_4_ and Al_2_(SO_4_)_3_ introduced a large amount of SO_4_^2−^, changing the solution equilibrium. The association effect of SO_4_^2−^ on H^+^ greatly weakened the acidity of the solution, which is conducive to the release of H^+^ from the titanium complex water and hydroxyl bridges, thus promoting the formation and extension of the Ti-O-Ti bond. Meanwhile, SO_4_^2−^ can connect two titanium complexes with a reversible bidentate chelate structure, shortening their distance from each other and promoting the connection of hydroxyl bridges and the formation of oxygen bridges. Al(SO_4_)_2_^−^ can form a similar structure, as well. These two mechanisms lead to more efficient hydrolysis and hydrated TiO_2_ with good dispersion.

## 4. Conclusions

In this work, hydrated TiO_2_ with good dispersion was prepared from a Mg/Al-bearing TiOSO_4_ solution with a low titanium concentration (0.8 mol·L^−1^ of TiOSO_4_) by the hydrothermal method, and the effect of MgSO_4_ and Al_2_(SO_4_)_3_ on the hydrolysis was investigated. The rapid and complete hydrolysis of TiOSO_4_ was achieved under the hydrothermal condition of 120 °C (hydrolysis time < 5 h, hydrolysis ratio ≈ 100%), and MgSO_4_ and Al_2_(SO_4_)_3_ accelerated the hydrolysis reaction and further shortened the hydrolysis time within 4 h. At the same time, the morphology and particle size of the hydrated TiO_2_ changed significantly and the particle size of the primary agglomerates increased from 40 nm to 140 nm. A thermodynamic equilibrium calculation indicated that MgSO_4_ and Al_2_(SO_4_)_3_ led to little change of [TiO^2+^] but a significant decrease of [H^+^], which is caused by the association effect of SO_4_^2−^ and favors the formation of Ti-O-Ti and hydrolysis of TiOSO_4_. In addition, Al(SO_4_)_2_^−^ and a reversible bidentate chelating structure of SO_4_^2−^ have been found that may synergistically promote hydroxyl bridge bonding and oxygen bridge formation. This study will provide new ideas and a theoretical basis for the recovery, development and high value utilization of TBFS.

## Figures and Tables

**Table 1:** Chemical composition of TBFS and the Mg/Al-bearing TiOSO_4_ solution.

TBFS	Component	TiO_2_	SiO_2_	CaO	Al_2_O_3_	MgO	Fe
	Content (wt%)	21~25	22~26	22~29	16~19	7~9	0.2~0.4
Mg/Al-bearing TiOSO_4_ solution	Component	TiOSO_4_	MgSO_4_		Al_2_(SO_4_)_3_	H_2_SO_4_	
	Concentration (mol·L^−1^)	0.8	0.5		0.4	1.0	

## Data Availability

No new data were created.

## References

[1] Fan G., Dang J., Lv X., Hu M. (2018). Effect of basicity on the crystallization behavior of TiO_2_-CaO-SiO_2_ ternary system slag. Crystengcomm.

[2] Wang L., Liu W., Hu J., Liu Q., Yue H., Liang B., Zhang G., Luo D., Xie H., Li C. (2018). Indirect mineral carbonation of titanium-bearing blast furnace slag coupled with recovery of TiO_2_ and Al_2_O_3_. Chin. J. Chem. Eng..

[3] Fan G.Q., Wang M., Dang J., Zhang R., Lv Z.P., He W.C., Lv X.W. (2021). A novel recycling approach for efficient extraction of titanium from high-titanium-bearing blast furnace slag. Waste Manag..

[4] Wang H., Feng K., Sun Q. (2018). Effect of calcium carbonate on the preparation of glass ceramic foams from water-quenched titanium-bearing blast furnace slag and waste glass. Adv. Appl. Ceram..

[5] Yang Z., Yang F., Yi M., Xiang L. (2021). Estimation of Reaction Heat in Ti-Bearing Blast Furnace Slag—Sulfuric Acid System Based on Mechanical Mixture Model. Min. Metall. Explor..

[6] Zhou H.-L., Feng K.-Q., Chen C.-H., Yan Z.-D. (2018). Influence of CeO_2_ addition on the preparation of foamed glass-ceramics from high-titanium blast furnace slag. Int. J. Miner. Met. Mater..

[7] Thompson T.L., Yates J.T. (2006). Surface science studies of the photoactivation of TiO_2_ new photochemical processes. Chem. Rev..

[8] Zhong B., Xue T., Zhao L., Zhao H., Qi T., Chen W. (2014). Preparation of Ti-enriched slag from V-bearing titanomagnetite by two-stage hydrochloric acid leaching route. Sep. Purif. Technol..

[9] Li Z.H., Wang Z.C., Li G. (2016). Preparation of nano-titanium dioxide from ilmenite using sulfuric acid-decomposition by liquid phase method. Powder Technol..

[10] Zhen Y.-L., Zhang G.-H., Chou K.-C. (2016). Carbothermic Reduction of Titanium-Bearing Blast Furnace Slag. High Temp. Mater. Process..

[11] Jalava J.P. (1992). Precipitation and Properties of Titania Pigments in the Sulfate Process. 1. Preparation of the Liquor and Effects of Iron(II) in Isoviscous Liquor. Ind. Eng. Chem. Res..

[12] Grzmil B.U., Grela D., Kic B. (2008). Hydrolysis of titanium sulphate compounds. Chem. Pap..

[13] Grzmil B., Grela D., Kic B. (2009). Formation of hydrated titanium dioxide from seeded titanyl sulphate solution. Chem. Pap..

[14] Tian C.X. (2020). Effects of Structural Factors of Hydrated TiO_2_ on Rutile TiO_2_ Pigment Preparation via Short Sulfate Process. Sci. Rep..

[15] Tian C.X., Huang S.H., Yang Y. (2013). Anatase TiO_2_ white pigment production from unenriched industrial titanyl sulfate solution via short sulfate process. Dye. Pigment..

[16] Zhang W., Ou C.R., Yuan Z.C. (2017). Precipitation and growth behaviour of metatitanic acid particles from titanium sulfate solution. Powder Technol..

[17] Xin W.H., Zhao Z.D., Yang X.H., Cheng Y.Z., Sun F.Z., Feng L. (2019). A Facile Preparation Strategy for TiO_2_ Spheres by Direct Hydrolysis. Chemnanomat.

[18] Tian M., Liu Y.H., Wang W.J., Zhao W., Chen D.S., Wang L.N., Zhao H.X., Meng F.C., Zhen Y.L., Hu Z.Y. (2020). Mechanism of synthesis of anatase TiO_2_ pigment from low concentration of titanyl sulfuric-chloric acid solution under hydrothermal hydrolysis. J. Chin. Chem. Soc. Taip..

[19] Wan X.B., Shi J.J., Qiu Y.C., Chen M., Li J.Z., Liu C.S., Taskinen P., Jokilaakso A. (2021). The effect of 15 wt% Al_2_O_3_ addition on the equilibrium phase relations of CaO-SiO_2_-TiO_2_ system at 1400 °C in air. Ceram. Int..

[20] Ma R.M., Chen Y.H., Yang Y., Wu Z.X., Bao Y.J., Li N., Li J.P. (2021). Facile hydrothermal deposition of octahedral-shaped Cu_2_O crystallites on bamboo veneer for efficient degradation of organic aqueous solution. Vacuum.

[21] Alli U., McCarthy K., Baragau I.-A., Power N.P., Morgan D.J., Dunn S., Killian S., Kennedy T., Kellici S. (2022). In-situ continuous hydrothermal synthesis of TiO_2_ nanoparticles on conductive N-doped MXene nanosheets for binder-free Li-ion battery anodes. Chem. Eng. J..

[22] Arlina A., Ter T.P., Ameram N., Najwa M.N., Norhafifi A., Norsyazleen M., Shaari A. (2022). Effect of heating times on the structural and optical properties of Al-doped ZnO via hydrothermal method. Mater. Today Proc..

[23] Nakamoto K., Fujita J., Tanaka S., Kobayashi M. (1957). Infrared Spectra of Metallic Complexes. IV. Comparison of the Infrared Spectra of Unidentate and Bidentate Metallic Complexes. J. Am. Chem. Soc..

[24] Navarrete J., Lopez T., Gomez R., Figueras F. (1996). Surface Acidity of Sulfated TiO_2_-SiO_2_ Sol-Gels. Langmuir.

[25] Li H.X., Li G.S., Zhu J., Wan Y. (2005). Preparation of an active SO_4_^2−^/TiO_2_ photocatalyst for phenol degradation under supercritical conditions. J. Mol. Catal. A Chem..

[26] Speight J.G. (2017). Lange’s Handbook of Chemistry.

[27] Bond G.C., Flamerz S. (1989). Structure and reactivity of titania-supported oxides: IV. Characterisation of dried vanadia/titania catalyst precursors. Appl. Catal..

[28] Stypula B., Stoch J. (1994). The characterization of passive films on chromium electrodes by XPS. Corros. Sci..

[29] Bierla A., Fromentin G., Minfray C., Martin J.-M., Le Mogne T., Genet N. (2012). Mechanical and physico-chemical study of sulfur additives effect in milling of high strength steel. Wear.

[30] Tian M., Liu Y.H., Wang L.N., Chen D.S., Zhao H.X., Meng F.C., Zhen Y.L., Qi T. (2021). Role of aluminum salt on thermal hydrolysis of titanyl sulfuric-chloric mixture acid solution. J. Mater. Res. Technol..

